# Aboveground Whitefly Infestation-Mediated Reshaping of the Root Microbiota

**DOI:** 10.3389/fmicb.2016.01314

**Published:** 2016-09-07

**Authors:** Hyun G. Kong, Byung K. Kim, Geun C. Song, Soohyun Lee, Choong-Min Ryu

**Affiliations:** ^1^Molecular Phytobacteriology Laboratory, Super-Bacteria Research Center, Korea Research Institute of Bioscience and BiotechnologyDaejeon, South Korea; ^2^Omics Pia Co. Ltd.Daejeon, South Korea

**Keywords:** bacterial community, rhizosphere, pyrosequencing, whitefly infestation, pepper, PGPR, *Pseudomonas*, microbiota

## Abstract

Plants respond to various types of herbivore and pathogen attack using well-developed defensive machinery designed for self-protection. Infestation from phloem-sucking insects such as whitefly and aphid on plant leaves was previously shown to influence both the saprophytic and pathogenic bacterial community in the plant rhizosphere. However, the modulation of the root microbial community by plants following *insect* infestation has been largely unexplored. Only limited studies of culture-dependent bacterial diversity caused by whitefly and aphid have been conducted. In this study, to obtain a complete picture of the belowground microbiome community, we performed high-speed and high-throughput next-generation sequencing. We sampled the rhizosphere soils of pepper seedlings at 0, 1, and 2 weeks after whitefly infestation versus the water control. We amplified a partial 16S ribosomal RNA gene (V1–V3 region) by polymerase chain reaction with specific primers. Our analysis revealed that whitefly infestation reshaped the overall microbiota structure compared to that of the control rhizosphere, even after 1 week of infestation. Examination of the relative abundance distributions of microbes demonstrated that whitefly infestation shifted the proteobacterial groups at week 2. Intriguingly, the population of Pseudomonadales of the class Gammaproteobacteria significantly increased after 2 weeks of whitefly infestation, and the fluorescent *Pseudomonas* spp. recruited to the rhizosphere were confirmed to exhibit insect-killing capacity. Additionally, three taxa, including *Caulobacteraceae*, *Enterobacteriaceae*, and *Flavobacteriaceae*, and three genera, including *Achromobacter*, *Janthinobacterium*, and *Stenotrophomonas*, were the most abundant bacterial groups in the whitefly infested plant rhizosphere. Our results indicate that whitefly infestation leads to the recruitment of specific groups of rhizosphere bacteria by the plant, which confer beneficial traits to the host plant. This study provides a new framework for investigating how aboveground insect feeding modulates the belowground microbiome.

## Introduction

Insects and plants have been interacting and co-evolving over the past 0.4 billion years. Under natural conditions, insects have several beneficial effects on plants, including protection from herbivores and help with pollination, while the plants provide a habitat and food for the insects (Panda and Khush, 1995). However, herbivore infestation can in some cases lead to the death of the plant. To protect themselves from insect infestation, plants have developed genetic and chemical defense mechanisms such as indirect defense via insect-derived plant volatiles (Birkett et al., 2003) and the production of toxic metabolites (Baldwin, 2001; Howe and Jander, 2008). At the same time, these organisms have established elaborate and varied relationships with microbes such as bacteria (Sugio et al., 2015). A growing body of studies on insect-plant-microbe interactions has broadened our knowledge of plant-derived modulation of microbe diversity to help plants survive under attack from insect pests (Pangesti et al., 2013).

Plant-insect-microbe interactions can be classified into two categories: microbial mediation of plant-insect interactions and insect mediation of plant-microbe interactions (Pineda et al., 2010, 2013; Biere and Bennett, 2013; Fu and Dong, 2013; Pangesti et al., 2013; Lazebnik et al., 2014; Sugio et al., 2015). Microbes influence plant-insect interactions by suppressing or enhancing infestation of the plant by herbivores. In this type of interaction, root colonization by the beneficial rhizobacterium *Azospirillum brasilense* provides insect resistance to corn plants and elicits the suppression of infestation by corn rootworm (*Diabrotica speciosa*) by increasing the emissions of *(E)*-*B*-caryophyllene in corn roots (Santos et al., 2014). Similarly, the presence of the plant growth-promoting rhizobacterium (PGPR) *Bacillus subtilis* leads to retarded development of whitefly (*Bemisia tabaci*) in tomato plants (Valenzuela-Soto et al., 2010). By contrast, the root application of certain soil bacteria enhances herbivore infestation by modulating plant immune signaling (Groen et al., 2013; Lazebnik et al., 2014). Pre-inoculation of *Pseudomonas fluorescens* WCS417r on the tomato root system increases the survivability of the nymph stages of whitefly (*B. tabaci*) by reducing the efficiency of defense responses related to the jasmonic acid (JA)-pathway (Shavit et al., 2013). In addition, the prior infection of *Pseudomonas syringae* on *Arabidopsis* leaves reduces plant resistance to cabbage looper (*Trichoplusia ni*) by enhancing ethylene signaling, thereby antagonizing salicylic acid (SA) signaling, which confers plant immunity to the target insect (Groen et al., 2013). Herbivores also modulate microbial behavior and community structure through regulating plant physiology and defense systems (Gehring and Bennett, 2009; Lakshmanan et al., 2012; Tack and Dicke, 2013). The belowground herbivorous insect *Agriotes lineatus* L. negatively affects the composition of fungal communities in the ragwort (*Jacobaea vulgaris*) rhizosphere (Kostenko et al., 2012). More specifically, infestation by the belowground insect wireworm (*Agriotes lineatus* L.) leads to the accumulation of the major plant defense compounds pyrrolizidine alkaloids in ragwort plants and reduces the levels of the pathogenic fungus *Fusarium oxysporum* in roots (Bezemer et al., 2013). By contrast, feeding by western corn rootworm larvae (*Diabrotica virgifera virgifera*) increases the density of the bacterial and fungal communities in maize (*Zea mays* L.) roots. Of all the members of the bacterial community whose populations increase in the rhizosphere due to insect infestation, the greatest increase occurs in *Acinetobacter calcoaceticus* (Dematheis et al., 2012). Even though recent studies have broadened our knowledge of plant-insect-microbe interactions, the effects of aboveground insect infestation on changes in commensal microbial communities were unknown until 2011.

In 2011, new information was obtained about how plants orchestrate resistance against the soil-borne pathogen *Ralstonia solanacearum* when whitefly (*Bemisia tabaci* Genn.) feeds on the leaf tissue of pepper (Yang et al., 2011). More intriguingly, whitefly infestation increases the populations of Gram-positive bacteria in the root zone known as the rhizosphere. These bacteria have beneficial effects on plants (Kloepper et al., 2004). Gram-positive *Bacillus* spp. act as a biological trigger to elicit plant systemic defense against subsequent whitefly infestation under field conditions (Murphy et al., 2000). Similarly, aphids, which like whitefly are sap-sucking insects, alter the population densities of *B. subtilis* GB03, as well as the Gram-negative bacterium *P. fluorescens* Pf-5, in the pepper rhizosphere (Lee et al., 2012). However, studies of insect-mediated changes in the populations of root-associated bacteria are limited due to their use of culture-dependent methodology. Analyses of variations in bacterial density due to whitefly or aphid infestation have traditionally been based on culture-dependent methods, but the diverse results obtained using molecular techniques suggest that reliance on culture-based approaches has led to an underestimation of bacterial diversity in the rhizosphere, which hampers estimation of the microbial diversity of plant rhizosphere microbiomes (Torsvik et al., 2002). To elucidate the functions of the altered bacterial populations, more sophisticated methods are needed to measure bacterial diversity.

**FIGURE 1:**
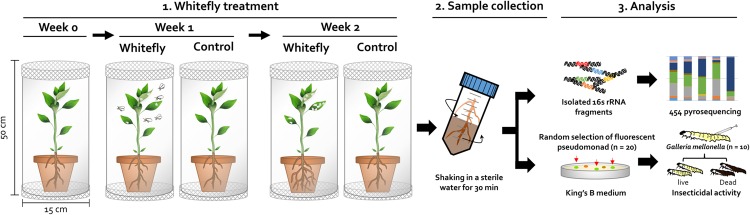
**Overview of workflow for investigating the microbial community in whitefly-infested pepper plants.** (1) *Whitefly treatment*: two-weeks-old pepper seedlings were treated with an average of 18 whitefly adults for 2 weeks in a plastic cylinder. (2) *Sample collection*: the root system was collected at 0, 1, and 2 week after whitefly infestation. The bacteria were then separated by shaking in SDW for 30 min. (3) *Analysis*: to investigate bacterial diversity, PCR-based 454 pyrosequencing (culture independent techniques) was employed after extraction of 16S rRNA from rhizosphere bacteria. To assess the insecticidal capacity of randomly selected pseudomonads, a killing assay with a model insect *Galleria mellonella* was conducted with 2 μL of bacterial suspension (OD_600_ = 1.0). *G. mellonella* mortality was measured at 24 h after inoculation at 30°C.

Recently, the microbial diversity in the rhizosphere was investigated by a culture-independent method based on amplified rRNA sequences from environmental samples (Smalla et al., 2001; Kirk et al., 2005; Inceoglu et al., 2013). Pyrosequencing technologies are culture-independent methods based on the principle of sequencing by synthesis, enabling the systematic culture-independent investigation of the plant rhizosphere microbiome (Chaparro et al., 2014; Bulgarelli et al., 2015; van der Voort et al., 2016). Such techniques can reveal the profiles of complex microbial taxonomic structures and specific bacterial communities in various plants such as rice, maize, oat, and wheat (Uroz et al., 2010; Knief et al., 2012; Turner et al., 2013). The rhizosphere soil, a narrow zone surrounding plant roots, contains dense populations of microbes (Hartmann et al., 2008; Mendes et al., 2011). The rhizosphere provides nutrients to the microbial community and influences bacterial activity and diversity, while the bacterial community in the rhizosphere is influenced by plant species, root exudates, plant age, and fungal diseases (McSpadden Gardener and Weller, 2001; Kowalchuk et al., 2002; Haichar et al., 2008; Mendes et al., 2011; Berendsen et al., 2012; Lundberg et al., 2012). A recent study demonstrated that the *Arabidopsis thaliana* rhizosphere contained different bacterial communities from those of bulk soil, as revealed by pyrosequencing (Lundberg et al., 2012; Inceoglu et al., 2013; Bulgarelli et al., 2015). The populations of Comamonadaceae, Flavobacteriaceae, Rhizobiaceae, Actinobacteria, and Proteobacteria were enriched in the *A. thaliana* rhizosphere, which was influenced by plant genotype, plant growth, and soil type (Lundberg et al., 2012; Bulgarelli et al., 2015). Several studies based on culture-dependent and -independent procedures show that great bacterial diversity exists in the rhizosphere (Bulgarelli et al., 2012, 2015; Lundberg et al., 2012; Chaparro et al., 2014). However, the rhizosphere bacterial communities of insect-infested plants are poorly understood. In this preliminary study, we performed next-generation sequencing (NGS) using the 454-pyrosequencing platform to evaluate the structure of the rhizosphere microbiome in the pepper plant rhizosphere in response to leaf infestation with whitefly. Collectively, the results of this study broaden our understanding of the role of the microbiome in insect–plant relations and the induction of systemic resistance, as well as the ecological value of the microbiome under natural conditions. The goal of this study was to provide new evidence that whitefly, a sucking insect that affects pepper, increases the populations of specific bacterial groups in the plant rhizosphere. Furthermore, we evaluated whether enriched *Pseudomonas* spp. have direct effects on insect herbivores (**Figure 1**). Investigating the effects of whitefly infestation on bacterial communities in the rhizosphere is important for understanding insect-plant-microbe interactions and their role in conferring beneficial traits to the host plant.

## Materials and Methods

### Plant Growth Conditions

Pepper (*Capsicum annuum* L. cv. Bukwang) was used as a model system in this study as described previously (Yang et al., 2011). *C. annuum* seeds were surface-sterilized with 6% sodium hypochlorite, washed four times with sterile distilled water (SWD), and germinated at 25–28°C for 3 days on 1/2 Murashige and Skoog medium supplemented with 0.6% (w/v) agar and 1.5% (w/v) sucrose. The seedlings were transplanted to natural soil collected from a pepper field located in Cheongwon-gun, Chungcheongbuk-do, South Korea (conducted in Cheongwon-gun, Chungcheongbuk-do, South Korea, 36° 35′ 32.27′′ North, 127° 30′ 34.75′′ East) in the KRIBB greenhouse facility, Daejeon, South Korea and grown at 25 ± 2°C for 2 weeks under controlled conditions in a growth chamber (12 h/12 h day/night cycle, c. 7000 L × light intensity). Each pepper plant was placed into an acrylic plastic cylinder (diameter = 15 cm, height = 50 cm, and thickness = 3 mm), and the top of the cylinder was covered with a nylon stocking as described previously (Yang et al., 2011; Kim et al., 2016).

### Whitefly Treatment

Whitefly (*B. tabaci*) were grown and maintained in the KRIBB greenhouse facility of Daejeon, South Korea in 2008–2010 as described previously (Yang et al., 2011; Park and Ryu, 2014). To investigate the effects of whitefly on the belowground bacterial microbiota in pepper plants, 2-week-old pepper plants were exposed to whitefly for 1 or 2 weeks (Whitefly at Week 1, WW1 and Whitefly at Week 2, WW2). The plants were exposed to an average of 18 ± 3.3 adult-stage whiteflies per pepper leaf (**Figure 1**). Control plants were grown without whitefly infestation at weeks 0, 1, and 2 (Control at Week 0, CW0, Control at Week 1, CW1, and Control at Week 2, CW2).

### Sampling and Amplification of the 16S rRNA Gene

To investigate the influence of whitefly infestation on belowground bacterial communities, 1 g of soil was sampled from the rhizospheres of whitefly-infested and control pepper plants at 1 and 2 weeks after treatment with whitefly, respectively (**Figure 1**). Plants grown in a growth chamber were removed from acrylic plastic cylinders. The roots from each sample were gently shaken to remove loosely attached soil, and tightly associated soil was separated from the roots by vigorous shaking in SDW for 30 min. The separated soil solution was centrifuged at 8,000 rpm for 10 min to collect rhizosphere soil containing microbiomes. The rhizosphere soil samples were stored at -80°C until use for microbial community analysis. Soil bacterial genomic DNA was extracted using a PowerSoil DNA kit (Mo Bio Laboratories, Solana Beach, CA, USA). Amplification of 16S rRNA and DNA sequencing were performed by OmicsPia, Co. Ltd (Daejeon, South Korea) according to the manufacturer’s instructions.

The 16S rRNA genes were amplified with universal primers (27F-GAGTTTGATCMTGGCTCAG and 518R-WTTACCGCGGCTGCTGG), which were used to amplify the V1–V3 regions of the bacterial 16S rRNA genes. To enable the separation of samples, specific barcode sequences were fused to the 5′ ends of the universal primers.

The 16S rRNA genes were amplified in a 50 μL (total volume) reaction mixture containing 1 μL of 100 ng/μL template DNA, 5 μL of 10X Ex Taq buffer, each deoxynucleoside triphosphate at a concentration of 2.5 μM, each primer of 20 nM, and 1.25 units of EX-Taq DNA polymerase (Takara Suzo, Co. Ltd, Tokyo, Japan). The polymerase chain reaction (PCR) was performed with a PCR thermocycler (Bio-Rad, Germany) under the following conditions: an initial denaturation step of 95°C for 5 min; 30 cycles consisting of denaturation at 95°C for 30 s, annealing at 55°C for 30 s, and extension at 72°C for 30 s; and a final extension step at 72°C for 7 min. The amplified PCR products were sequenced using a GS-FLX Titanium Pyrosequencer (454 Life Sciences, Branford, CT, USA) at OmicsPia, Co. Ltd.

### Pyrosequencing Analysis of Using the Mothur Pipeline

Amplicon reads of the partial 16S ribosomal RNA genes (V1–V3 regions) generated by the 454 GS FLX Titanium platform were initially trimmed for quality using the Pyrotrimmer program v1.1 (Oh et al., 2012). Bacterial 16S rRNA sequence data from the microbiota in the rhizosphere of pepper plants were processed through the mothur pipeline (Schloss et al., 2009). Reads were sorted into each sample based on their unique barcodes and were error-corrected using the PyroNoise algorithm. Chimeric sequences were filtered out using the UCHIME algorithm after the nearest alignment space termination based on the SILVA database (DeSantis et al., 2006; Edgar et al., 2011). High-quality controlled reads were taxonomically assigned using RDP classifier with a 0.8 confidence threshold. The reads were also used to determine diversity indices and unique sequences and to evaluate the abundance of observed operational taxonomic units (OTUs), which were clustered at 3% dissimilarity in each sample (Wang et al., 2007). Using these OTUs, construction of distance matrix and clustering were conducted using the mothur pipeline. Alpha diversity was estimated using various diversity and richness indices , such as the Shannon index, and Inverse Simpson index, abundance-based coverage estimators (ACEs), and Chao1 (a non-parametric richness estimator), which were calculated using mothur analyses (Schloss et al., 2009). For beta diversity analysis, principal coordinates analysis (PCoA) was conducted using the Bray–Curtis metric. The Bray–Curtis algorithm was used to calculate the distance between samples (Beals, 1984). PCoA was conducted using the Bray–Curtis metric. RDP LibCompare was used to estimate the probability of differences in the abundance of some observed phylogenetic taxa between samples. The pyrosequencing experiment at CW1 (Control at Week 1), CW2 (Control at Week 2), WW1 (Whitefly at Week 1), and WW2 (Whitefly at Week 2) was repeated at least twice.

### Quantification of Rhizosphere Fluorescent Pseudomonads

The population of bacteria on the roots was measured at 0, 1, and 2 weeks after whitefly exposure as described previously (Yang et al., 2011). In brief, whitefly-infested pepper roots were incubated in 30 mL of SWD for 30 min in a shaking incubator at 30°C. The population of root-colonizing *Pseudomonas* spp. was determined by plating on King’s B-agar medium (KB; 10 g proteose peptone No. 3, 1.5 g K_2_HPO_4_, 1.5 g MgSO_4_⋅7H_2_O, 10 mL glycerol, 20 g agar, and 1 L distilled water) (King et al., 1954). The pseudomonad population was calculated based on the number of fluorescent colonies under UV light irradiation (UVP, Inc., Upland, CA, USA) at 365 nm. The experiment was conducted using a completely randomized design with 10 replications. Twenty fluorescent colonies per treatment were randomly selected for further evaluation of insecticidal activity. The experiment was repeated at least twice with 10 biological replications.

### *Galleria mellonella* Killing Assay

Insecticidal activity analysis was performed with *Galleria mellonella* as described previously (Chung et al., 2016). Ten randomly chosen *G. mellonella* caterpillars were used for each selected bacterium in an experiment. Prior to inoculation, 20 of pre-selected pseudomonads per treatment as described above were adjusted to an optical density OD_600_ of 1.0 with phosphate-buffered saline (PBS). A 2 μl bacterial suspension was injected into the body cavity of each *G. mellonella* caterpillar using 10-μL Hamilton syringe (25-gauge, Hamilton, Co., Reno, NV, USA). After Injection, *G. mellonella* caterpillars were incubated in a growth chamber at 30°C to assess the number of dead caterpillars at 24 h after inoculation (**Figure 1**). The experiment was repeated at least twice with 20 biological replications.

### Statistical Analysis

Analysis of pyrosequencing data was performed with the R program (R Core Team, 2014) with the additional multcomp packages (Hothorn et al., 2008). Statistical analyses of experimental datasets were performed using commercial statistical software (JMP v5.0, SAS Institute, Inc., Cary, NC, USA). Significant effects of treatment were determined based on the magnitude of the *F*-value (*P* = 0.05). When a significant *F*-test was obtained, separation of means was accomplished using Fisher’s protected least significant difference (LSD) at *P* = 0.05.

## Results

### Plant Rhizosphere Bacterial Community Is Affected by Whitefly

To profile the belowground bacterial community, we amplified 16S rRNA genes in the rhizosphere using 12 pepper plants, including five control plants and seven plants whose leaves were infested with whitefly. A total of 341,009 reads were sorted by the Protrimmer program. After a de-replication step, 284,945 unique reads were obtained. After removing chimeric and chloroplastic sequences, 196,554 sequences were obtained for all samples.

After the reads were clustered into OTUs, those with sequence similarity >97% were discarded from the analysis, resulting in 23,596 OTUs (**Table 1**). A total of OTUs were obtained for the whitefly infested pepper rhizosphere, and microbial diversity analysis was performed based on species diversity and evenness index. The bacterial diversity of samples was estimated by the Shannon and Inverse Simpson metrics. Bacterial richness in whitefly-infested samples at week 2 (WW2) appeared to be significantly lower that of the control plant samples (CW0 and CW1, respectively; one-way ANOVA, *P* < 0.05). Chao1 and ACE metric, which are used for richness analysis, revealed similar patterns in the control and whitefly infested pepper rhizosphere (one-way ANOVA, *P* < 0.08; **Table 2**).

**Table 1:** Total number of reads, observed operational taxonomic units (OTUs), Good’s coverage, diversity index (Shannon’s and Inverse-Simpson index), and richness (Chao1 and ACE) for each sample measured based on a 3% dissimilarity cutoff.

Treatments	Reads	Observed OTUs	Good’s coverage	Shannon’s Index	Inverse-Simpson	Chao1	ACE
CW0	7886^b^	2914^a^	0.72^b^	6.7^a^	178.07^a^	10713^a^	22809^a^
CW1	10798^b^ 3347	2763^a^ 196	0.78^b^ 0.09	5.69^a^ 1.20	139.39^b^ 184.01	8818^a^ 3235	18179^a^ 7026
CW2	13863^b^ 3122	2182^b^ 294	0.875^ab^ 0.05	4.02^a^ 0.96	10.19^d^ 6.66	5092^ab^ 1557	11023^ab^ 3940
WW1	7549^b^ 132	1954^b^ 50	0.80^b^ 0.0	5.27^ab^ 0.02	20.19^c^ 1.05	7573^a^ 285	16213^a^ 126
WW2	24847^a^ 2793	1376^b^ 289	0.954^a^ 0.02	2.04^ab^ 0.47	2.36^d^ 0.54	2360^b^ 689	4657^b^ 1514

*Richness represents the number of observed unique operational taxonomic units (OTUs), which was estimated by the estimator Chao1 and the abundance-based coverage estimator (ACE). Diversity is indicated by the Shannon and Inverse Simpson indexes. Evenness is measured as the ratio of Shannon index to the number of observed OTUs. All data were expressed as the mean ± SEM. Statistical significance was calculated using a one-way ANOVA. Different letters such as a–d indicate significant difference based on LSD (P = 0.05).*

**Table 2:** Summary of the relationships between major taxa and genera.

	Taxa (significance value > 0.01)	Genus level
CW1 > WW1	Caulobacteraceae (*Brevundimonas, Asticcacaulis, and Phenylobacterium*)	*Massilia*
CW2 > WW2	Cytophagaceae (*Cytophaga, Flectobacillus, and Dyadobacter*)	-
	Oxalobacteraceae (*Massilia, Undibacterium, Naxibacter, and Herbaspirillum*)	-
	Xanthomonadaceae (*Rhodanobacter, Stenotrophomonas, Thermomonas, and Rudaea*)	-
	Paenibacillaceae (*Paenibacillus and Cohnella*)	-
CW1 < WW1	Microbacteriaceae (*Microbacterium and Leifsonia*)	*Ralstonia*
	Mycobacteriaceae (*Mycobacterium*)	*Sphingobium*
	Flavobacteriaceae (*Chryseobacterium*)	*Variovorax*
CW2 < WW2	Caulobacteraceae (*Brevundimonas*)	*Achromobacter*
	Enterobacteriaceae (*Escherichia/Shigella*)	*Janthinobacterium*
	Flavobacteriaceae (*Elizabethkingia*)	*Stenotrophomonas*

*The bacterial taxa and genus represent the different major populations at the different treatments. CW1 = Control at Week 1, CW2 = Control at Week 2, WW1 = Whitefly at Week 1, WW2 = Whitefly at Week 2.*

**FIGURE 2:**
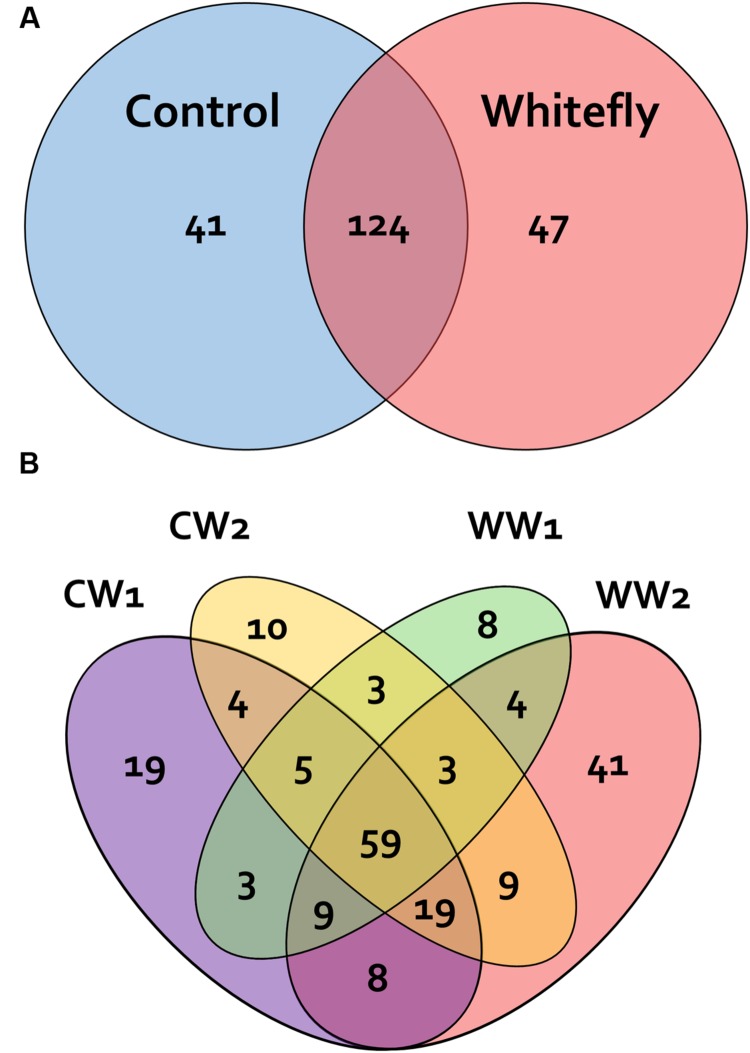
**Venn diagrams representing the number of unique and shared operational taxonomic units (OTUs 97% sequence similarity).** Diagrams comparing pyrosequencing results from the control and whitefly-infested pepper plant rhizosphere **(A)** and after different durations of whitefly infestation and the control **(B)**. OTUs are defined at 97% sequence similarity. The relative abundance of shared OTUs across all samples is shown in parentheses.

Among the total OTUs, 41 were exclusively detected in the control and 47 were exclusively detected in the whitefly infested plant rhizosphere. A total of 124 OTUs were shared with two other groups (**Figure 2A**). Furthermore, as observed in the Venn diagrams (**Figure 2B**), the samples at 2 weeks after whitefly infestation contained the highest number of endemic OTUs (41 OTUs).

### Structure of the Bacterial Community

**FIGURE 3:**
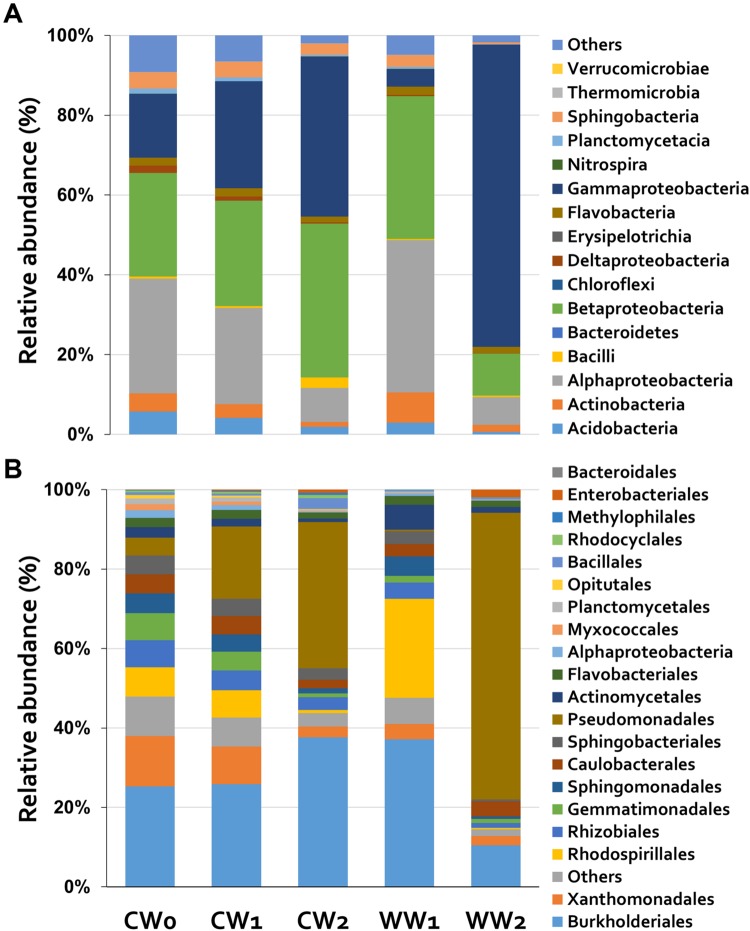
**Relative abundance (%) of rhizosphere bacteria.** The composition of the bacterial community in the rhizospheres of three control treatments (CW: control) and two whitefly-infested treatments (WW: Whitefly treatments). Numbers indicate exposure time to whitefly (CW0: time zero; CW1 and WW1: 1 week; CW2 and WW2: 2 weeks). The distribution of the different bacterial phyla is based on data obtained by 454 sequencing. Distribution of classes with relative abundance (>0.3% dissimilarity; **A**) and orders **(B)** in control and whitefly infestation samples.

We detected differentially abundant bacterial communities in the control versus whitefly infested plant rhizosphere. Bacterial community structure analysis at the class level showed that, in all samples, alpha-, beta-, and gammaproteobacteria were the major bacterial communities. However, the relative abundance of gammaproteobacteria was highest at 2 weeks after whitefly infestation (WW2; 76 ± 11%), whereas the abundance of alpha- (7 ± 4%) and betaproteobacteria (11 ± 4%) decreased (**Figure 3A**). At the order level, the abundance of the *Pseudomonadales* population (72 ± 12%) was higher at 2 weeks after whitefly infestation (WW2). By contrast, the populations of *Xanthomonadales* (13%), *Burkholderiales* (25%), and *Sphingomonadales* (5%) were larger in the control at the beginning of analysis (CW1; **Figure 3B**).

**FIGURE 4:**
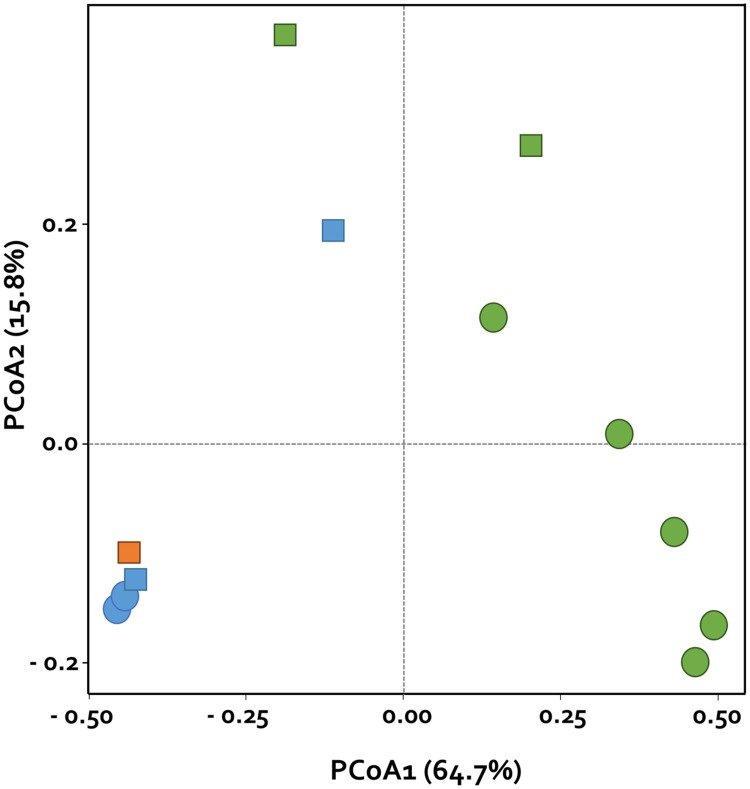
**Principal coordinates analysis (PCoA) based on Bray–Curtis distance matrix of bacterial community compositional structure in pepper plants.** Plant rhizosphere without (squares) and with (circles) whitefly infestation. Samples were taken at two different time points after whitefly infestation and are represented as follows: orange indicates time zero, blue indicates 1 week, and green indicates 2 weeks after whitefly infestation. PCoA1 (64.7%) and PCoA2 (15.8%) are the first and second principal coordinates, respectively.

Principal coordinates analysis based on the Bray–Curtis dissimilarity index revealed clear differences between the two groups at the genus level. The first two axes as PCoA explained 64.7 and 15.8% of the variation, respectively. In the whitefly infested samples, we observed closer clustering, and the distances between sampling times of the two groups were variable (**Figure 4**). The abundances of *Brevundimonas*, *Asticcacaulis*, and *Phenylobacterium* of the family Caulobacteraceae were higher among abundant OTUs in the control at 1 week after infestation (CW1), whereas the abundances of Microbacteriaceae (genus *Microbacterium* and *Leifsonia*), Mycobacteriaceae (genus *Mycobacterium*), and Flavobacteriaceae (genus *Chryseobacterium*) were higher in whitefly infested plants at 1 week after infestation (WW1). The abundance of *Rhodanobacter*, *Stenotrophomonas*, *Thermomonas*, and *Rudaea* of the family Xanthomonadaceae increased among abundant OTUs in the control at 2 weeks after infestation (CW2), whereas the abundances of *Escherichia/Shigella* of the family Enterobacteriaceae and *Elizabethkingia* from Flavobacteriaceae were higher in whitefly infested plants at 2 weeks after infestation (WW2; **Figure 4**).

### Analysis of Fluorescent Pseudomonad Abundance and Insecticidal Effect

**FIGURE 5:**
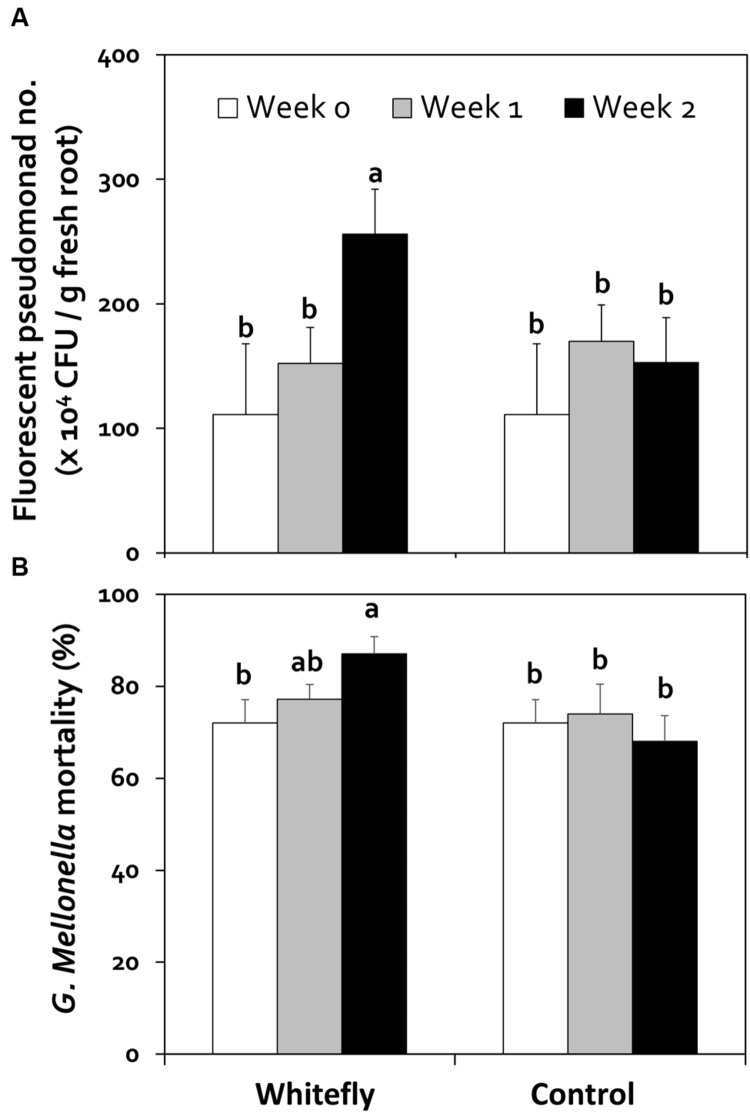
**Effects of whitefly infestation on fluorescent pseudomonad populations in the pepper rhizosphere and insecticidal activity. (A)** Fluorescent pseudomonad community in the rhizosphere. The population of pseudomonads was quantified by plating on King’s B medium at 0, 1, and 2 weeks after whitefly infestation (*n* = 10). The number of colony-forming units (CFUs) of fluorescent pseudomonads was determined under UV light and expressed per gram of root fresh weight. **(B)** Evaluation of *Galleria mellonella* mortality by *Pseudomonas* spp. (*n* = 20). *G. mellonella* mortality was evaluated for 24 h after injection of 2 μL *Pseudomonas* spp. suspension. *G. mellonella* caterpillars (*n* = 10) were incubated in a growth chamber at 30°C after Injection to assess the number of dead caterpillars. Values are mean ± SEM at ^∗^*P* < 0.05 according to the LSD test.

Assessment of the effects of whitefly infestation on the plant rhizosphere, specifically *Pseudomonas* spp. diversity against insect infestation, using a culture-based method on King’s B medium showed that whitefly-infested plants had significantly (*P* < 0.05, *n* = 10) higher fluorescent pseudomonad populations at 2 weeks than 1 week and the control plants, whereas the control plants at 1 and 2 weeks were similar (**Figure 5A**). To investigate the effects of *Pseudomonas* spp. on insect killing, we randomly selected 20 fluorescent colonies to assess insecticidal activity for each time period. To determine whether such differences in pathogenicity to selected fluorescence *Pseudomonas* spp. occurred between the whitefly-infested samples and the control, we inoculated *G. mellonella* caterpillars with these *Pseudomonas* spp. As shown in **Figure 5B**, *G. mellonella* mortality was significantly higher in caterpillars inoculated with *Pseudomonas* spp. isolates from pepper root after whitefly application than those of the control (*P* < 0.05; **Figure 5B**).

## Discussion

The recent analysis of plant-associated microbiomes represents a new horizon in botanical and agricultural research (Mendes et al., 2013). Previous studies examining the role of microbes in insect-plant-microbe tritrophic interactions were limited, as few utilized culture-independent 16S rRNA amplicon sequencing technology, also referred to as NGS. In the current study, we demonstrated that whitefly (*B. tabaci* Genn.) infestation of pepper plants modulates the rhizosphere bacterial community, leading to the enrichment of Pseudomonadales of the class Gammaproteobacteria, as determined using a NGS platform, 454 pyrosequencing, and a culture-based method. The results of pyrosequencing indicate that the bacterial diversity and evenness in the plant rhizosphere were influenced by whitefly infestation rather than by the sampling times of the plant rhizosphere (**Table 1**; **Figure 3**). However, in a study of *Arabidopsis*, the bacterial diversity and evenness in the microbiomes in the rhizosphere were found to be unrelated to plant developmental time point (Lundberg et al., 2012; Chaparro et al., 2014). This finding indicates that the rhizosphere sampling time does not have much of an effect on bacterial diversity. However, in the current study, the bacterial communities in the rhizospheres of whitefly infested plants exhibited slight differences in OTUs, diversity, and evenness at 2 weeks of whitefly infestation (WW2) compared to 1 week (WW1; **Table 1**). These results indicate that specific bacterial populations were recruited to the rhizosphere due to whitefly infestation.

An intersection of OTUs in each sample, when grouped by treatment or sampling time, was observed for 28.9% of the OTUs, which shared 97% sequence similarity and were shared between the whitefly infested plant and control plant rhizospheres (**Figure 2**). The shared OTUs represent essential microbial communities in the plant rhizosphere, whereas the endemic OTUs in WW2 might be helpful for the whitefly infested plants. PCoA also indicated that each sample was clustered according to whitefly infestation and sampling time (**Figure 4**). The results indicate that specific bacterial populations were affected in the changing bacterial community. A previous study indicated that the level of a specific bacterial population, i.e., Gram-positive bacteria, increased in the whitefly infested pepper rhizosphere compared to the control (Yang et al., 2011). However, the current study demonstrates that whitefly feeding on pepper leaves led to a significant increase in Gram-negative bacteria (**Figure 3**). These different results might be attributed to the different techniques used: in the previous study, bacterial colonies on artificial media were measured, while, in the present study, we detected the number of OTUs based on the presence of 16S rRNA in the rhizosphere. Taken together, our results more comprehensively reflect the bacterial community.

Based on comprehensive analysis of the essential or endemic OTUs, we estimated the relative abundances of members of the bacterial community. Our results show that the population of Pseudomonadales of the class Gammaproteobacteria significantly increased after 2 weeks of whitefly infestation, as revealed through both culture-dependent and -independent methods (**Figure 3**). We propose three possible scenarios to explain these results: (1) plants secrete root exudates specifically to attract *Pseudomonas* spp. following whitefly infestation. This idea is supported by our current and previous finding that the variation in rhizosphere microbes between WW1 and WW2 may be influenced by the altered secretion of root exudates and the expression of plant signaling genes (**Figure 5A**). Previously, we found that whitefly infestation induces four transporter genes, including the genes encoding ATP-binding cassette (ABC) transporter, peptide transporter, zinc transporter, and phosphate transporter, as well as two auxin-responsive genes, which can increase the root biomass and help recruit microbes in whitefly infested plants (Yang et al., 2011; Park and Ryu, 2014). In addition, recent genome sequencing of *Pseudomonas* spp. of diverse origins revealed that they contain insecticidal gene clusters such as *Fit, TccC*, and *Mcf* (Kupferschmied et al., 2013; Bruto et al., 2014; Flury et al., 2016). A study of natural variation across *Pseudomonas* spp. and field application of specific strains of *Pseudomonas* spp. demonstrated their insect-killing capacity. The *Pseudomonas* spp. are also distributed in both the phyllosphere and rhizosphere, indicating that plants indirectly protect themselves against subsequent whitefly infestation. (2) A recent study demonstrated that plant phenolic compounds such as anthocyanin and salicylic acid (SA) are major secreted products of plants when aphids attack their leaves (Park and Ryu, 2014; Song et al., 2015). Previously 6 μg/mL SA secretion by whitefly infestation was shown to be effective against soil microbiota (Song et al., 2015). Interestingly, most *Pseudomonas* spp. are resistant to SA, while other Gram-negative bacteria are sensitive, leading to the elimination of the SA-sensitive bacterial population (Ramos, 2004). (3) Finally, the accumulated SA in the rhizosphere leads to an increase in the remaining SA-resistant population, such as *Pseudomonas* spp. In addition, researchers have long investigated the beneficial effects of Pseudomonadales, typically including *Pseudomonas* spp. The *Pseudomonas* spp. include a large number of species that provide benefits for plants, such as plant growth promotion and biocontrol (Raaijmakers et al., 1995; McSpadden Gardener and Weller, 2001). Similarly, the populations of Burkholderiales of the class Betaproteobacteria and Rhodospirillales of the class Alphaproteobacteria significantly increased at 1 week of infestation (**Figure 3B**). Overall, these results indicate that rhizosphere microbiota react rapidly to whitefly infestation, leading to the dominance of different bacterial taxa over time. The reason that whitefly mediated changes in plant physiology lead to changes in the rhizosphere microbiome is still largely unknown. One possible explanation is that the recruitment of *Pseudomonas* spp. helps protect plants against possible subsequent attack from soil-borne insect pests. Many species of insect pests complete their life cycles from the larval stage in the soil to aboveground infestation.

A more detailed classification of the bacterial community at the genus level revealed that the populations of *Achromobacter*, *Janthinobacterium*, and *Stenotrophomonas* were altered with whitefly infestation, suggesting that whitefly infested plants specifically select microbes (**Table 2**). *Achromobacter* promotes the growth of oilseed-rape (*Brassica napus*), wheat (*Triticum aestivum*), and *Brassica juncea* by improving the absorption of nitrogen, producing indole acetic acid (IAA), and functioning in phosphate solubilization (Bertrand et al., 2000; Jha and Kumar, 2009; Ma et al., 2009). These findings are also in agreement with the previous observation (Park and Ryu, 2014) that plant auxin-related genes are upregulated at 1 week after whitefly infestation (Park and Ryu, 2014). Moreover, *Stenotrophomonas* strains can produce IAA *in vitro*, which influences root development (Suckstorff and Berg, 2003), and indole-dependent priming increases the levels of plant stress hormones such as jasmonate–isoleucine conjugate and abscisic acid (Erb et al., 2015). Under whitefly infestation, these hormones may elicit systemic resistance against bacterial pathogens and abiotic stress (Yang et al., 2011; Park et al., 2015; Song et al., 2015). Therefore, our results indicate that whitefly infestation enriches the population sizes of specific bacteria, including IAA-producing bacteria, which play an important role in plant growth both directly and indirectly, by priming plants for defense responses.

## Conclusion

This is the first report demonstrating the transition of belowground microbial communities elicited by aboveground insect herbivores. Many studies using various ecological systems demonstrate that insect infestation aboveground systemically affects plant defense mechanisms. The effects of insect infestation on plant rhizosphere microbes have only recently begun to be understood. Moreover, the interactions of insect-plant-microbes remain poorly understood. Revealing the composition of the microbiome community in the whitefly infested plant rhizosphere and unraveling the underlying mechanisms will increase our understanding of the effects of insects and plants on the rhizosphere environment. Out of all communities of the microbiome, members of the Gammaproteobacterial group, including *Pseudomonas* spp. containing the insecticidal capacity, are the major enriched communities that respond to whitefly feeding. Moreover, the NGS technique and culture-base procedure employed in this study shed light on the novel insect-plant-microbe tritrophic interaction, thus representing a promising development. A more detailed study of the role of the recruited *Pseudomonas* spp. and other enriched bacterial genera in the rhizosphere of pepper plants infested by phloem-sucking insects should be performed in the near future. In addition, the ecological meaning behind the current results must also be determined to apply this information to pest management programs.

## Author Contributions

HGK: performed both *in vitro* screening of pseudomonad and insecticidal activity, analyzed the microbiome data, and wrote the paper. BKK: performed the bioinformatics analysis. GCS and SL: DNA isolation from plant rhizosphere and performed the experiments. CMR: Supervise the design of experiments and the processes. HGK, BKK, GCS, SL, and CMR read and approved the final version of the manuscript.

## Conflict of Interest Statement

The authors declare that the research was conducted in the absence of any commercial or financial relationships that could be construed as a potential conflict of interest.
